# Distinct structural variants and repeat landscape shape the genomes of the ancient grapes Aglianico and Falanghina

**DOI:** 10.1186/s12870-024-04778-2

**Published:** 2024-02-06

**Authors:** Riccardo Aversano, Marina Iovene, Salvatore Esposito, Alberto L’Abbate, Clizia Villano, Ermanno Di Serio, Maria Francesca Cardone, Carlo Bergamini, Riccardo Aiese Cigliano, Vincenzo D’Amelia, Luigi Frusciante, Domenico Carputo

**Affiliations:** 1 Department of Agricultural Sciences, University of Naples Federico II, Portici, Italy; 2 Institute of Biosciences and Bioresources, National Research Council of Italy (CNR-IBBR), Portici, Italy; 3 Research Centre for Cereal and Industrial Crops, Council for Agricultural Research and Economics (CREA-CI), Foggia, Italy; 4 Institute of Biomembranes, Bioenergetics, and Molecular Biotechnologies, National Research Council (IBIOM-CNR), Bari, Italy; 5 Research Centre for Viticulture and Enology, Council for Agricultural Research and Economics (CREA-VE), Turi, Italy; 6 Sequentia Biotech, Carrer de València, Barcelona, Spain

**Keywords:** Resequencing, Variant calling, Satellite DNA, Repetitive elements, *Vitis vinifera* L

## Abstract

Mounting evidence recognizes structural variations (SVs) and repetitive DNA sequences as crucial players in shaping the existing grape phenotypic diversity at intra- and inter-species levels. To deepen our understanding on the abundance, diversity, and distribution of SVs and repetitive DNAs, including transposable elements (TEs) and tandemly repeated satellite DNA (satDNAs), we re-sequenced the genomes of the ancient grapes Aglianico and Falanghina. The analysis of large copy number variants (CNVs) detected candidate polymorphic genes that are involved in the enological features of these varieties. In a comparative analysis of Aglianico and Falanghina sequences with 21 publicly available genomes of cultivated grapes, we provided a genome-wide annotation of grape TEs at the lineage level. We disclosed that at least two main clusters of grape cultivars could be identified based on the TEs content. Multiple TEs families appeared either significantly enriched or depleted. In addition, in silico and cytological analyses provided evidence for a diverse chromosomal distribution of several satellite repeats between Aglianico, Falanghina, and other grapes. Overall, our data further improved our understanding of the intricate grape diversity held by two Italian traditional varieties, unveiling a pool of unique candidate genes never so far exploited in breeding for improved fruit quality.

**Supplementary Information:**

The online version contains supplementary material available at 10.1186/s12870-024-04778-2.

## Introduction

The cultivated grape (*Vitis vinifera* subsp. *vinifera*, 2n = 2x = 38) germplasm contains a considerable genetic complexity expressed by a great wealth of varieties [1]. In the last decade (re-)sequencing of hundreds of *V. vinifera* genomes has shed lights on such diversity revealing that along with single nucleotide polymorphisms (SNPs), structural variations (SVs) are at base of existing grape variability at both intra- and inter-species levels [2–9]. SVs are either the presence/absence (PAVs) or differences in the copy number (CNVs) of DNA sequences among genomes, and other chromosomal rearrangements such as inversions and translocations [10, 11]. It has been estimated that SVs may be present in 5–15% of the grape genome, encompassing hundreds of genes [4, 5, 8]. The functional impact of such variations is unexplored. However, several studies pointed to the potential for SVs to contribute to the phenotypic diversity between grapes because these genomic regions are enriched in genes involved in noteworthy traits, such as defense response, biosynthesis of aromatic compounds, and embryo development [4, 5, 8].

Additional genomic diversity comes from the repetitive DNA sequences, which are either dispersed, mainly as a result of transposons and retrotransposon activity, or arranged in tandem such as the satellite repeats. Transposable elements (TEs) are very diverse depending on how they are transposed. These include DNA transposons (classified as Class II TE) that are mobilized directly via “cut and paste” mechanisms and long terminal repeat (LTR) retrotransposons (Class I TE) that move via a “copy and paste” pathway. Satellite DNA (satDNA) repeats consists of arrays, up to megabases in length, of tandemly arranged repeated units (monomers) that are predominantly concentrated in the heterochromatic regions of the chromosomes [12, 13]. In grape, TEs are well-known mediators of genetic plasticity and modulators of biological diversity for their affecting the activity of adjacent genes [14–16]. For example, the Gret1 (Grapevine Retrotransposon 1) retroelement played a pivotal role in generating berry color variations in grapevine clones [17]; a transposable element of the hAT family caused the multiplication and branching of flower meristems in a clone of Carignan [18]; and the Mila-flb inverted-repeat transposable element was responsible for the fleshless berry (flb) somatic variant in the variety Ugni Blanc [19]. However, while TEs are important contributors to genomic variability and somatic mutation in grape, few studies have focused on their genome-wide characterization and annotation at the lineage level in grapes [20, 21]. In addition, these studies were based on few cultivars, which provided a limited perspective on the extent of the diversity in repeat composition at the intra-species level.

Similarly, to TEs, satDNA, being a fast-evolving portion of the eukaryotic genome (for a review, see [22, 23]), may contribute to the genomic differentiation between closely related species and even at intraspecific level [24–28]. Some satDNAs, such as those located at telomeres and nucleolar organizer regions, are involved in essential functions [22, 23]. Moreover, even satDNA arrays not associated with such regions can exert several effects at genomic and evolutionary levels, for example by reducing either the expression of neighboring genes or the local recombination [28, 29]. SatDNAs are a major cause of the large gaps left in the chromosome assemblies because of the challenges to assemble arrays of nearly identical sequences. To fill those gaps, different approaches have been used, based on a combination of next-generation sequencing with appropriate bioinformatic tools, molecular cytogenetics, and more recently also with long read sequencing [22, 30, 31]. Currently, there is no genome-wide profile of the grape satDNA, and the knowledge about its abundance, chromosomal distribution and intra-species diversity in grapes is limited [32–34].

Southern Italy is recognized as the oldest wine growing area of Italy [35]. The wine grapes Aglianico and Falanghina are known to be the oldest varieties of Southern Italy and are still cultivated to produce high-quality red and white wines, respectively. Together with Strinto Porcino, Visparola and Montonico Bianco, Aglianico (AGL) is one of the founding varieties of the traditional cultivated grapes of the South-Western Italy [1]. It is a later-maturing grape characterized by a high content of total flavonols and anthocyanins, with a notable presence of quercetin-3-O-glucoside, malvidin-3-O-glucoside, and petunidin-3-O-glucoside [36–39]. Falanghina (FAL), instead, stands out for its amino acids and terpenoid contents, contributing to the typical wines fruity and slightly floral aromas [40, 41]. Genomic data on Falanghina are scanty despite its diffusion at national level (more than 3.000 ha) and wine appreciation (Agroqualità, 2020). The genomic footprints underlying such biochemical traits of AGL and FAL have not been investigated yet. In this study, we re-sequenced the Aglianico and Falanghina genomes through Illumina technology to deepen our understanding of their genomic variation in SNPs, SVs and repetitive DNA sequence composition. By leveraging AGL and FAL genomic data, along with a set of publicly available genomes of *vinifera* varieties, we provided a comprehensive characterization of the grape repeatome and gained insight into their repeat composition. Overall, the data generated further improved our understanding of the diversity held by two major and traditional Italian grapes.

## Materials and methods

### Plant material, DNA isolation and sequencing

This study was performed on *V. vinifera* cv. Aglianico (AGL) biotype Taburno (clone Ampelos TEA22) and cv. Falanghina (FAL) del Beneventano (clone Ampelos EVA1), both grafted onto rootstock 1103 Paulsen – *V. berlandieri* x *V. rupestris* (clone ISV1). Samples were collected from a nine-year-old vineyard (41°13′43.00″ N, 14° 33′ 37.56″ E, 145 m a.s.l., Castelvenere) during the 2017 growing season. Leaves from three different plants were harvested, pooled and immediately frozen in liquid nitrogen and then stored at − 80 °C until extraction. High-quality genomic DNA was extracted from three biological replicates as described by Japelaghi et al. [42], with few modifications, and an equimolar pool of DNA was used for sequencing. Two libraries were sequenced on Illumina Hi-seq1000 (Illumina, San Diego, CA, USA) by Genomix4life s.r.l. (Italy) yielding a total of 180 M reads per sample.

### Reference-guided assembly and genomes annotation

Raw Illumina reads were processed with Trimmomatic (v. 0.33) to remove adapter/primer sequences and trim 5′ and 3′ -end bases (minimum quality 35, minimum length 35 bp). Quality of trimmed sequences was checked using FastQC (v0.11.3; http://www.bioinformatics.babraham.ac.uk/projects/fastqc/ (October 2017)). Reference-guided assembly and genome annotation were performed following the strategy reported by Tranchida-Lombardo and colleagues [43] with some modifications (see Methods S1). To test whether the distribution of variants per chromosome was random, a Chi-square test was applied using the observed number of variants against the number of variants that would be expected from a random distribution of the variants based only on the length of each chromosome (i.e., expected number = Average Number of Variants per Kbp * Length of the Chromosome). To reannotate the Aglianico and Falanghina genomes we used the RNA-Seq recently produced by Villano et al. [38] on a panel of six tissues/developmental stages (pulp and skin at pre-*veraison*, *veraison* and harvesting) of the same AGL and FAL clones. SNPs identified in AGL and FAL during the iterative variant calling were functionally annotated with respect to the reference genome annotation with SNPEff (10.4161/fly.19695, October 2017). Gene Ontology Enrichment Analysis (GOEA) was performed with in-house scripts as described in Methods S1.

### Read-depth analysis and digital CGH

We performed a modified whole-genome shotgun detection (WSSD) analysis [44] in the two genomes. AGL and FAL raw reads were aligned to the reference genome employing the mrFAST aligner (95% sequence identity). Absolute copy number (CN) was calculated using mrCaNaVaR (http://mrcanavar.sourceforge.net), considering non-overlapping unmasked windows of 1kbp (KbUS). 5 KbUS sliding windows were used to predict duplications and deletions. Segmental duplications (SDs) and deletions were defined as regions with at least five consecutive windows with a CN > 2.5 and CN < 1.5, respectively [44, 45]. An in silico digital comparative genome hybridization (CGH) was performed to detect CN variations among the AGL and FAL sequenced genomes [46]. The estimated CN of each window for each variety was compared with the CN of the same window in the reference genome. The log2 ratio (L2R) of that comparison was calculated and we considered regions > 10 Kbp with L2R > 0.25 and L2R < 0.25 as amplified or deleted, respectively. The identified copy number variations were inspected to define which were common or variety-specific.

### Variants analysis of genes involved in the biosynthesis of secondary metabolites

The key genes of the pathways of terpenes, green leaf volatiles (GLVs), branched-chain amino acids (BCAAs), and phenylpropanoids were identified as reported in Esposito et al. [47] and Villano et al., [48]. The proteins used as queries to search for amino acid orthologs in Aglianico and Falanghina genomes were obtained either from *A. thaliana* or *V. vinifera* as reported in Methods S1. The orthologs in Aglianico and Falanghina were searched using HMMER [49] as reported by Esposito et al. [50] for all gene families. Only sequences with an e-value lower than 10^−5^ and an identity higher than 85% with the selected gene were regarded as putative and further analyzed. The full-length candidate proteins were manually confirmed by checking the domain using the NCBI search domain online tool [51].

### Transposable elements annotation

The repeated fraction was evaluated by graph-based clustering of repetitive elements in unassembled reads using the RepeatExplorer2 Web server [52, 53]. Twenty-one grapevine genotypes were selected from Magris et al. [9] (Methods S1) to capture the highest genotypic diversity in the available dataset and to compare the results with the Aglianico and Falanghina of the present study. Raw reads were obtained through the “European Nucleotide Archive” (EBI) database. Seqtk (https://github.com/lh3/seqtk) was used to extract 1 M random reads (seed 100) from each sample. Adapter removing and read quality analysis were performed with Trimmomatic (v0.39) [54] to trim bases with a quality score (QS) < 20, remove reads < 100 nt, and cut reads to 100 nt to obtain a subset of high-quality reads of the same length for each sample (100 nt). Finally, roughly 250,000 high quality random reads of each sample (corresponding to 0.01 × of their genome size) were analyzed as reported by Novak et al. [53] and detailed in the Methods S1. REXdb database (Viridiplantae version 3.0) was used as reference database of transposable elements domains (http://repeatexplorer.org/?page_id=918).

### Cytological validation of selected satellites

The distribution of selected putative satellites on grape chromosomes was assessed by fluorescence in situ hybridization (FISH) as previously described [55, 56]. Oligonucleotide probes and PCR primers for FISH are provided in Data S1. Along with AGL and FAL samples, grape variety Greco Bianco (GRC) was also included in this analysis. Immature inflorescences of AGL, FAL and GRC were fixed in 3:1 (100% ethanol: glacial acetic acid) Carnoy’s solution. Mitotic and meiotic chromosomes were prepared as previously described [55, 56], with minor modifications (Methods S1). Images were captured with a DFC365 FX CCD camera and LAS AF software using a Leica DM6000B epifluorescence microscope (Leica Microsystems). The final contrast of the images was adjusted in Adobe Photoshop.

## Results

### Genome annotation and detection of intervarietal small variations

**Fig. 1:**
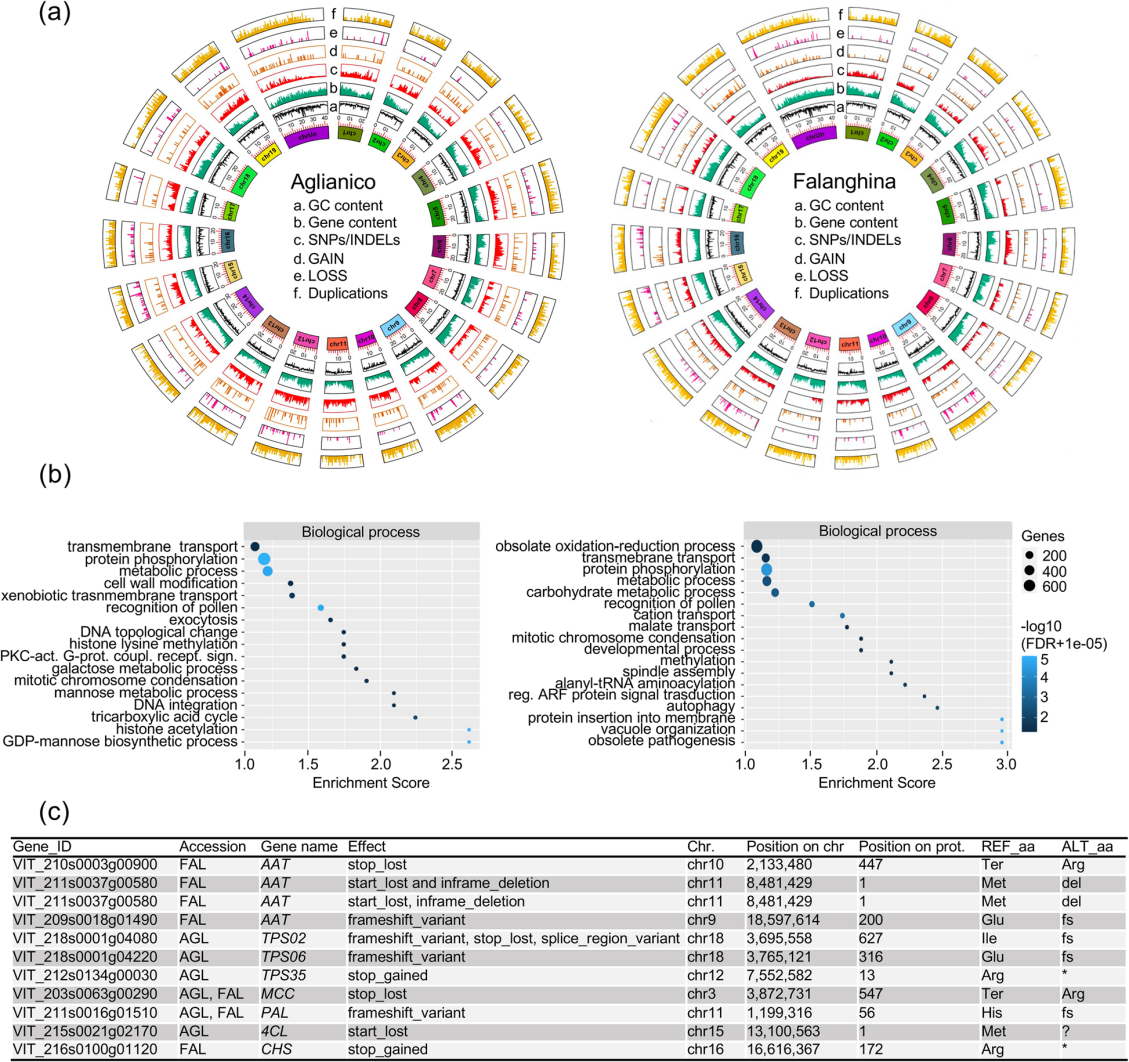
**a** SNP distribution and abundance in Aglianico (left) and Falanghina (right) genomes relative to the Pinot Noir reference genome (PN40024_12X.v2). From the inner circle the plots show: chromosome size; GC content in 100 Kbp bins; gene content in 100 Kbp bins; SNPs/INDELs in 100 Kbp bins; GAIN in 100 Kbp bins; LOSS in 100 Kbp bins; Duplications in 100 Kbp bins. **b** Gene Ontology Enrichment Analysis (GOEA) results performed on AGL (left) and FAL (right) genes harboring missense mutations and genes including polymorphisms altering CDS length. Enriched terms related to biological process are reported. **c** Summary of high-impact variants found in AGL and FAL within enzyme-coding genes involved in the terpenoids (*TPS*, *terpene synthase*), GLVs (*MCC*, *Methylcrotonyl-CoA carboxylase*), BCAA (*AAT*, *alcohol acetyltransferase*) and phenylpropanoid (*PAL*, *phenylalanine ammonia-lyase*; *4CL*, *4-coumarate: CoA ligase; CHS, chalcone synthase*) biosynthetic pathways. * stands for stop codon

The Reconstructor pipeline [43, 57] allowed us to generate reference genomes of roughly 483 Mb for both AGL and FAL, in agreement with the estimated size of other grapevine genomes [58–60]. Both genomes were organized in 19 chromosomes (coverage of 30x), of which Chr14 resulted as the longest and Chr17 the shortest in both genomes (Table S1). The genome annotation pipeline using RNAseq data yielded 31,142 genes (encoding for 53,889 transcripts) in AGL and 31,544 genes (encoding for 56,622 transcripts) in FAL (Table S1). Direct comparisons with the Pinot Noir reference genome (PN40024) [58, 61] revealed that 91.5% (1,591,170), 4.3% (74,133) and 4.2% (73,900) of the AGL polymorphic sites were SNPs, deletions, and insertions, respectively (Table S1). Similar percentages were found in FAL, although a smaller number of deletions (54,390) and insertions (55,770) was observed compared to AGL (Table S1). During the last step of the Reconstructor pipeline 42 and 22 novel contigs were identified and successfully placed within the chromosomes of AGL and FA, respectively (Table S1). Although small variants were distributed among all chromosomes of both genomes (Fig. 1a), we noticed that their distribution was not random (Chi-square test *p*-value < 0.05) both for individual chromosomes and genome wide (Table S2).

We observed an enrichment of variants in some chromosomes (on Chr06, Chr11, Chr12, Chr17 and Chr19 in AGL, and Chr06 and Chr10 in FAL), with an average of 4.42 variants per Kbp. In addition, Chr12 and Chr19 showed a relatively lower rate of variants per Kbp in FAL (0.88 and 0.65 variants/Kbp) with respect to AGL (4.7 and 4.11 variants/Kbp) (Table S2). Approximately 1400 genes harboring potential disruptive effects were identified in both genomes. Gene Ontology Enrichment Analysis (GOEA) indicated the predominance of “histone acetylation” and “GDP-mannose biosynthetic process” as the most abundant terms related to the biological process in AGL, whereas “protein insertion into membrane”, “vacuole organization” and “obsolete pathogenesis” were the most enriched in FAL (Fig. 1b, Figure S1). Since the position of SNPs may influence the functionality of the encoded proteins, we sought polymorphisms within annotated genes, posing particular attention to those involved in the biosynthesis of key enological compounds of both varieties, namely green leaf volatiles (GLVs), branched-chain amino acids (BCAAs), terpenoids and phenylpropanoids (Tables S3–S7). The orthology analysis of GLVs-related sequences found 67 (in AGL) and 59 (in FAL) genes encoding *lipoxygenases* (*LOX*), *hydroperoxide lyase* (*HPL*), *alcohol dehydrogenase* (*ADH*) and *alcohol acetyltransferase* (*AAT*) (Tables S3 and S4). The largest gene family was the AAT, with 41 and 37 members in AGL and FAL, respectively. The genetic variant annotation and functional effect prediction highlighted six high-impact changes in FAL AAT homologs. Among them, only VIT_209s0018g01490.2 exhibited a distinct pattern of expression between the two varieties, with increased expression observed in all comparisons for AGL and decreased expression in the pulp comparison for FAL (Fig. 1c, Table S4).

Concerning the BCAA pathway, we identified 36 (in AGL) and 37 (in FAL) genes corresponding to 22 different enzymes (Tables S3 and S5). In two different *Methylcrotonyl-CoA carboxylase* (*MCC*) isoforms, a stop-loss variant was predicted in AGL and FAL with no consequences at the transcriptional level (Fig. 1c). In the terpenoids pathway, we identified 83 (in AGL) and 84 (in FAL) sequences homolog to 15 different enzymes (Tables S3 and S6). High-impact variants were found only in Aglianico *terpene synthase* (*TPS*) *02*, *TPS07* and *TPS35* (Fig. 1c). In AGL, TPS35 was found to have a frameshift variant and a stop gained, and it did not show differential expression. However, in FAL, TPS35 was identified as a differentially expressed gene that was overexpressed in the skin comparisons. Concerning the phenylpropanoid pathway, 80 (in AGL) and 75 (in FAL) genes were identified (Tables S3 and S7). Each variety exhibited two high impact variants in a *Phenylalanine Ammonia-Lyase* (PAL) and in a *Chalcone Synthase* (CHS). Only in FAL, the CHS gene with a stop gained variant was differentially expressed.

### Structural variations (SVs)

Absolute copy number (CN) values calculation disclosed very similar levels of duplications (CN > 2.5, corresponding to around 31% of their genomes) and deletions (CN < 1.5, roughly 1% of their genomes) in AGL and FAL. We found 4112 duplicated regions that were shared between the two varieties, and 70% of them contained genes (Table S8). In silico digital CGH analysis enabled the identification of CN polymorphisms in AGL and FAL compared to the Pinot genome reference (PN40024). AGL possesses 356 (2.1% of the genome), and 362 (2.9% of the genome) gained and deleted regions, respectively, ranging from 10 to 776 Kbp. Similarly, in FAL, we identified 351 gains (2% of the genome) and 316 losses (2.3% of the genome), spanning between 10 and 235 Kbp (Table S9). Then, we looked at the polymorphic regions (hereafter Copy Number Variant Regions, CNVRs) shared between AGL and FAL and checked their gene content. In AGL and FAL we found 163 CNVRs with increased CN (gained CNVRs) compared to the Pinot reference and 149 with a diminished CN (loss CNVRs) (Table S10 and S11). Among the gained CNVRs, 59 showed CN values almost double in AGL and FAL with respect to those observed in PN40024, and 15 had a CN > 50. Similarly, among the loss-shared CNVRs, 135 were found in duplicated regions in PN40024, with about half of CN in FAL and AGL. According to the publicly available gene ontology (GO) functional annotation, 292 (in the gained CNVRs) and 293 (in the loss CNVRs) genes were mainly linked to ion transport and DNA replication processes, respectively (Table S10 and S11). The most polymorphic genes were related to defense (NBS-LRR), stress and signaling mechanisms, which are gene families already known to be duplicated. Interestingly, genes potentially involved in the formation of floral aroma occurred in CNVRs located on Chr5, Chr9, Chr19 (e.g., three genes involved in the monoterpenoids biosynthesis) and ChrUnknown (e.g., 3-hydroxyisobutyryl-CoA hydrolase-encoding genes) (Table S10). Similarly, members of the cinnamyl alcohol dehydrogenase gene family, linked to the phenylpropanoid biosynthesis and metabolism, were found in duplicated regions on Chr13. Finally, homologs of the NADH-dehydrogenase cytochromes gene families related to the photosynthesis and oxidative phosphorylation pathways were discovered among the most polymorphic CNVRs, with CN values higher than 50 in many cases (Table S10). By contrast, among the shared loss CNVRs, we found a region on Chr18 containing three UDP-glucose:3-deoxyanthocyanidin 5-O-glucosyltransferase (dA5GT) with CN values in AGL and FAL halved with respect to that found in Pinot Noir (CN = 6). Also, genes involved in glycan structure biosynthesis (e.g., exostosin family protein) were mapped in two shared loss CNVRs on Chr11 (Table S11).

### AGL and FAL interspersed repeats and comparative analysis of their repeatome landscape

Individual clustering analysis of AGL and FAL (~ 250,000 random reads/sample) with RepeatExplorer2 [53] revealed that both varieties shared a similar amount of repetitive sequences (~ 40%). Both DNA repertoires were composed of different families belonging to class I (retroelements), class II (DNA transposons) elements, rDNA, and satellite DNA repeats, although a small fraction (~ 7%) remained unclassified in both genomes (Table S12). Proportions of DNA transposons, which included four different families (Enspm_CACTA, hAT, MuDR Mutator and PIF Harbinger), accounted for roughly 2% in both AGL and FAL. Among them, MuDR Mutator and hAT (~ 1%) showed a higher representation compared to CACTA and PIF Harbinger elements (~ 0.16%). Among non-LTR retrotransposons, LINEs accounted for roughly 2% in both individuals, whereas the pararetrovirus group was scarce (less than 0.2% of the genomes). The LTR retrotransposons (Ty1/Copia and Ty3/Gypsy) abounded (~ 23%), with a slight predominance of the Ty3/Gypsy superfamily (13%) over Ty1/Copia elements (10%). At the lineage level, seven different Ty3/Gypsy (Athila, Ogre, Retand, Tekay, Reina, Galadriel, and CRM) and eight Ty1/Copia lineages (Ale, Angela, Bianca, Ikeros, Ivana, SIRE, TAR, Tork) were identified in both AGL and FAL, although a small fraction of Ty1/Copia remained unclassified (< 2%). Among Ty3/Gypsy lineages, Athila retroelements were the most represented in both genomes, showing genomic proportions over 5.5% (Table S12), whereas the *Ale* lineage was the most represented among the Ty1/Copia elements (2.9% on average).

**Fig. 2:**
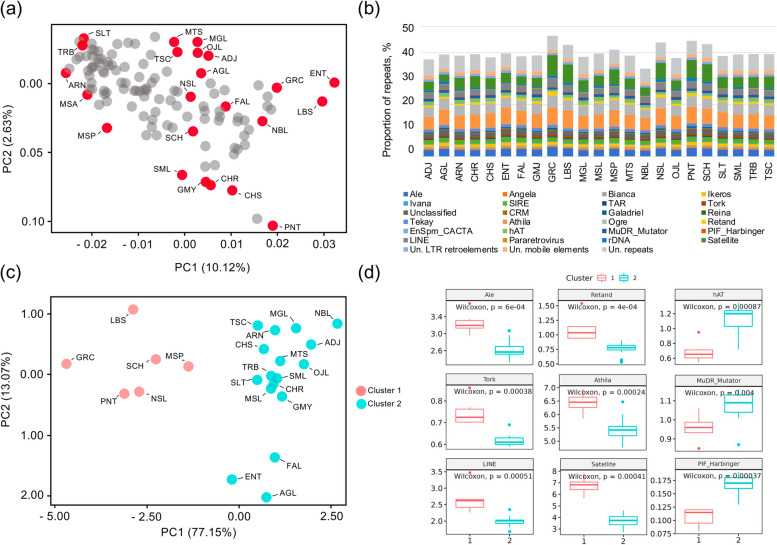
**a** Principal Component Analysis (PCA) of 23 *V. vinifera* whole-genome resequenced genotypes by [9]. The samples selected in this study to perform the repeatome comparative analysis are red-dotted, namely Adjaruli Tetri (ADJ), Aglianico (AGL), Airen (ARN), Chardonnay (CHR), Chasselas Blanc (CHS), Enantio (ENT), Falanghina (FAL), Gamay Noir (GMY), Greco Bianco (GRC), Lambrusco di Sorbara (LBS), Mgaloblishvili (MGL), Mtsvane Kachuri (MTS), Muscat of Alexandria (MSL), Muscat Petits Grains Blanc (MSP), Nebbiolo (NBL), Nosiola (NSL), Ojaleshi (OJL), Pinot Noir (PNT), Schiava Gentile (SCH), Semillon (SML), Sultanina (SLT), Terbash (TRB), Tschvediansis Tetra (TSC). **b** Proportion of DNA repetitive sequences identified. (C) PCA of different grape cultivars based on their repeats landscape. Clustering was performed with K-means and colors were assigned according to the cluster. **c** Boxplots showing the most significant and enriched families of repeats in clusters 1 and 2 of PCA shown in (**c**). Wilcoxon test results are shown for each repeat class

A comparative analysis of the whole repeatome landscape has never been performed among the *V. vinifera* genomes. Therefore, from the whole-genome sequencing data produced by [9], we selected 21 samples representative of the genetic diversity of the cultivated germplasm (Fig. 2a) to perform a comparative clustering analysis.

The repetitive fraction ranged among the accessions from 33.8% in Nebbiolo to 46.4% in Greco Bianco (Table S12). Results from comparative clustering confirmed the repetitive landscape observed in AGL and FAL (Fig. 2b). Among Ty3/Gypsy*,* Athila and Ogre were confirmed as the two most represented lineages in *V. vinifera*. The genome fraction of the former elements ranged from 4.8% in Nebbbiolo to 7% in Lambrusco di Sorbara, whereas the latter accounted for roughly 4% in all samples. Among the Ty1/Copia lineages, Ale was the most abundant, in agreement with the individual clustering analysis carried out in AGL and FAL. Proportions of DNA transposons (Enspm_CACTA, hAT, MuDR Mutator and PIF Harbinger) ranged between 1.7% in Greco Bianco to 2.9% in Mgaloblishvili (Table S12). Finally, satDNA varied in terms of genome representation from 2.7% in AGL to more than 7.0% in Greco Bianco (see below). We then conducted a PCA to summarize the genomic differentiation in the relative repeat contents among the whole set of 23 varieties (Fig. 2c). We found that the varieties were well differentiated on the first principal component (PC1) in two main clusters, with cluster one composed by Greco Bianco, Pinot Noir, Nosiola, Schiava gentile, Muscat à Petits Grains Blancs and cluster 2 by the remaining accessions (Fig. 2d). However, FAL, AGL and Enantio were separated from the rest of the cluster 2 to which they are assigned mostly on the PC2, suggesting that these cultivars had a partly distinct repeat composition from the rest of the cluster. When the relative abundances of the different repeats were compared between the two clusters, it appeared that 19 families were significantly different (Wilcoxon test, *p*-value <= 0.05, Figure S2). The most significant and enriched families in Cluster 1 were Ale, Tork, Athila, Retand, Line and Satellite. On the other hand, Cluster 2 was enriched for hAT, MuDR_Mutator and PIF_Harbinger (Fig. 2d).

### Identification of satellite repeats and cytological validation

Using the RepeatExplorer2 pipeline, we detected 16 putative satellite repeat clusters in AGL and FAL that were shared with the other grape varieties considered in this study. SatDNA fraction accounted for about 4.5% of the grape genome, with a range between ~ 3%, as in AGL, FAL and Ojaleshi, to > 7%, in Greco Bianco and Lambrusco di Sorbara (Fig. 2). The satellite clusters differed for their estimated genomic abundance (from > 2% of VvSat1 to < 0.1% of VvSat214), monomer length (from 42 nt to 994 nt) and A/T content (from 48 to 77%). Here, we focused on potential satellites with a typical monomer length of ~ 100–300 nt, as well as on three families with longer monomers of 677–994 nt (Table S13).

VvSat1, the most abundant satDNA in each variety, had a monomer length of 107 nt and average A/T content of 51%. VvSat1 shared pairwise sequence similarities with other repeat clusters with similar, shorter (56, 78 and 83 nt) and longer motifs (293 nt) (Figure S3), suggesting that these repeats likely represent variants of a single satellite (super-)family. Differences in the amounts of VvSat1 and its related-repeats were responsible for the intraspecific variability in the SatDNA content of grapes. Indeed, varieties with higher estimates of SatDNA, such as Greco Bianco and Pinot, contributed at least 3 times more reads to the VvSat1-related clusters than the varieties with lower SatDNA estimates, such as AGL and FAL. Interestingly, the VvSat1 consensus monomer shared high sequence identity with a candidate centromeric motif of grapes [33, 34]. Fluorescence in situ hybridization (FISH) using a VvSat1-related repeat (VvSat21) labeled most AGL and FAL chromosomes in a single location, that is, their primary constrictions (Figure S4A,B). Signal intensity varied among chromosomes, with weak or non-detectable signals on about 10 centromeres (Figure S4). For comparison, we also analyzed a clone of Greco Bianco, because of the relatively higher SatDNA content of its genome (see above). The FISH pattern of VvSat1-related repeats in Greco Bianco resembled that of AGL or FAL, with no apparent differences in their abundance and/or distribution (Figure S4C1-C3). Another putative satellite, VvSat85, with a typical monomer length of 187 nt (Table S13), had an estimated genomic proportion of about one tenth of the VvSat1 repeats. VvSat85 monomers contained an almost perfect palindrome of 43 nt (Data S1), a frequent feature of satellite repeats (for a review [23]). FISH using VvSat85 probe generated interstitial and subterminal signals on about ten chromosomes in both AGL and FAL with main signals overlapping with heterochromatic bands (Figure S5).

**Fig. 3:**
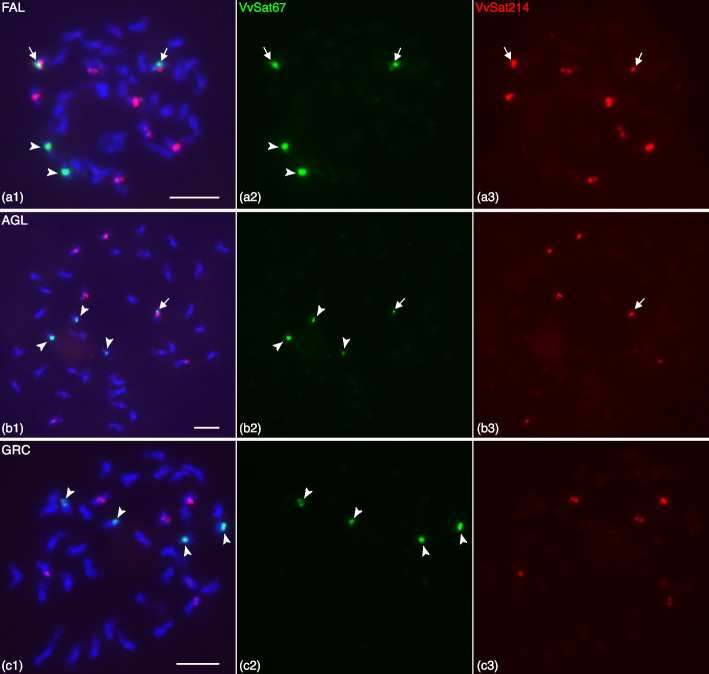
Fluorescence in situ hybridization (FISH) of VvSat67 (green signals) and VvSat214 (red) repeats on the mitotic metaphase chromosomes (stained in blue) of different grape accessions: (A1–3) Falanghina (FAL); (B1–3) Aglianico (AGL); (C1–3) Greco Bianco (GRC). Arrowheads point to VvSat67 signals (greens) that are located independently from VvSat214, on different chromosomes. Arrows (in A1–3 and B1–3) point to VvSat67 and VvSat214 signals that co-localize on the same chromosome(s). Scale bars = 5 μm

Concerning the satellites with longer monomers, our analysis detected three potential satellites, namely VvSat67, VvSat214 and VvSat158, with monomers of 994, 964, and 677 nt, respectively (Table S13). Similarity searches against the reference genomes of Pinot Noir and Cabernet Sauvignon supported their tandem organization (Methods S1; Tables S14 and S15). VvSat67 mapped on the pseudomolecules of Chr15 and 17 of both reference genomes (Tables S14 and S15). The distribution of VvSat214 and VvSat158 differed between the two reference genomes, indicating potential differences in their chromosomal distribution and number of sites in diverse grapes (Methods S1; Tables S14 and S15). For example, VvSat214 monomers were located on Chr10, 11, 15 and 16 of Pinot, whereas, on Cabernet, it mapped on three pseudomolecules of the haplotype 1 (Chr10, 11 and 19) and four pseudomolecules of haplotype 2 (Chr10, 11, 15, and 16, Tables S14 and S15). To provide experimental support to the in silico mapping, we performed FISH using VvSat67, VvSat214 and VvSat158 repeats on the mitotic chromosomes of AGL and FAL, as well as Greco Bianco for comparison. VvSat67 generated four signals on four somatic chromosomes in each variety (Fig. 3). These signals overlapped with pericentric heterochromatic regions (Figure S6) and had slightly different strengths (Fig. 3). FISH using VvSat214 revealed a different distribution among the varieties, with signals on eight, six and five chromosomes of FAL, AGL and Greco Bianco, respectively (Fig. 3, Figure S7).

In FAL, two chromosomes with VvSat214 repeats also carried VvSat67 (Figure 3A1-A3, Figure S7A1-A3), whereas in AGL only one of the chromosomes with a VvSat214 site also carried a VvSat67 (Figure 3B1-B3, Figure S7B1-B3). In Greco Bianco, none of the VvSat214 sites co-localized with VvSat67 (Figure 3C1-C3, Figure S7C1-C3). Based on the in silico mapping results (see above), the chromosome(s) carrying both VvSat67 and VvSat214 in AGL and FAL likely correspond to Chr15.

**Fig. 4:**
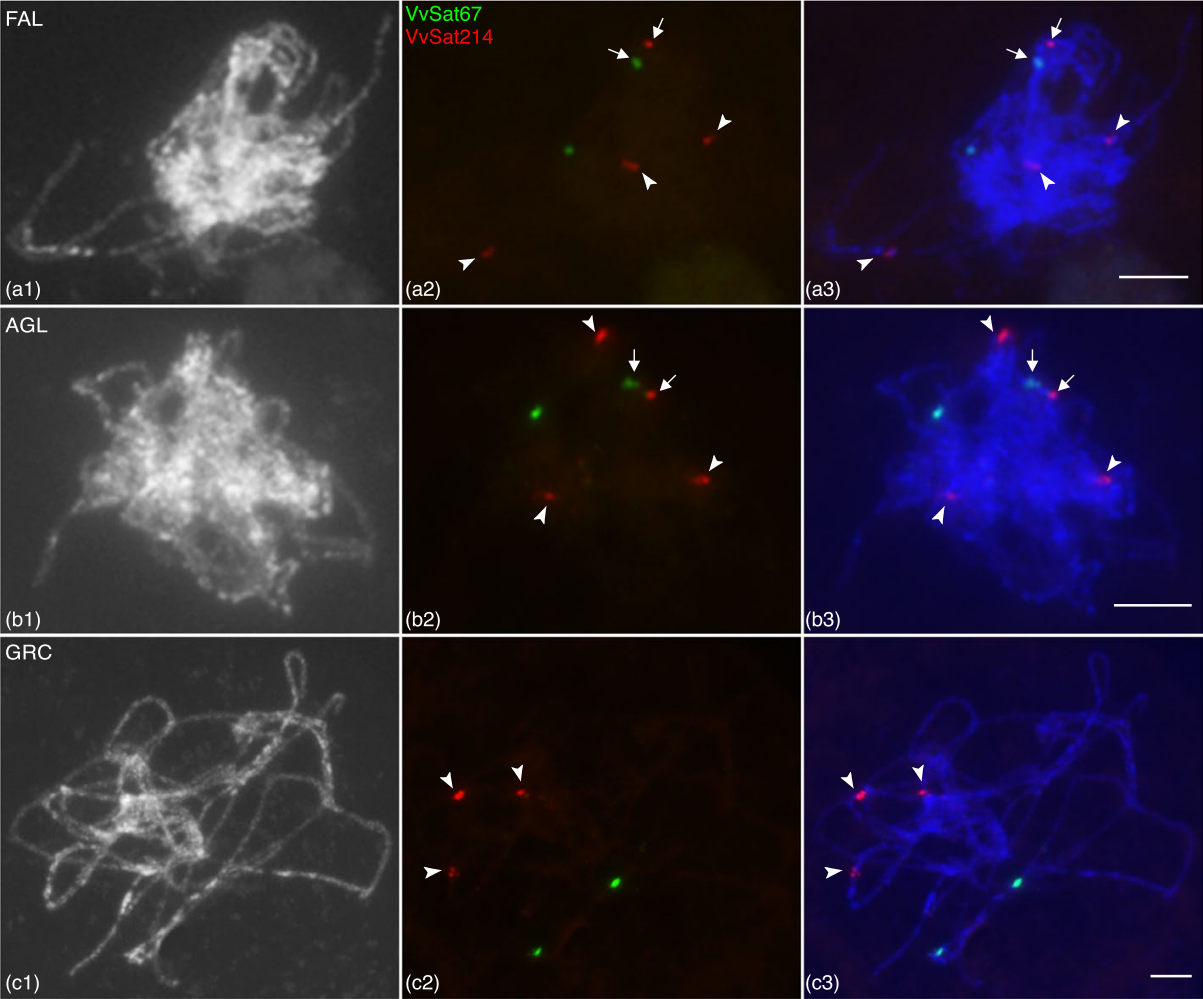
FISH mapping of VvSat67 and VvSat214 repeats on the meiotic pachytene chromosome of Falanghina (FAL), Aglianico (AGL) and Greco Bianco (GRC) grapes. Black and white images of DAPI stained pachytene chromosomes of (A1) FAL; (B1) AGL; and (B1) GRC. Middle column: Signals derived from VvSat67 (green) and VvSat214 (red). Third column: Merged images. Arrowheads point to VvSat214 signals (in red) that are located on a different chromosome from VvSat67 signals (green). Arrows (in A1–3 and B1–3) point to VvSat67 and VvSat214 signals that co-localize on the same chromosome. Scale bars = 5 μm

As expected, in FAL, the number of FISH signals at the meiotic pachytene stage was half of those detected on mitotic chromosomes (two and four signals for VvSat67 and VvSat214, respectively) (Figure 4A1-A3). FISH using VvSat67 produced the expected two signals on the pachytene chromosomes of AGL and Greco Bianco (Fig. 4b, c). However, VvSat214 generated four and three signals on the pachytene chromosomes of AGL and Greco Bianco, respectively (Fig. 4), indicating that some VvSat214 loci did not pair with one another and were hemizygous. As for VvSat158, which also had a variable number of sites based on the in silico mapping results, FISH using VvSat158 on somatic metaphase spreads generated major signals on five chromosomes in AGL and Greco Bianco, and six chromosomes in FAL (Figure S8). These findings supported that VvSat214 and VvSat158 sites have a variable number and distribution in grapes, including hemizygous sites in some varieties as detected in AGL.

## Discussion

### Identification of copy number variants in secondary metabolism genes with enological significance

Around 30% of FAL and AGL genomes were enriched in highly similar segmental duplications, confirming the highly plastic nature of grapevine genomes. Given the importance of SVs in shaping genome structure and driving gene evolution, we sought genes with different copy number in both genomes with respect to the reference (PN40024). We found 718 CNVs in AGL and 667 in FAL, many of them mapping in regions already duplicated in the reference genome. This confirms that SVs are hotspots for CNVs formation [5, 44, 51, 62]. Around 70% of these regions contain genes that therefore result polymorphic. This finding enforces the hypothesis that genes subjected to CNV are potential candidates for causing phenotypic differences between varieties, as previously reported in many plant species [63–69] and grapevine [5]. Among the most polymorphic genes encompassed within AGL and FAL CNVs, we found members related to signaling, stress mechanisms and involved in photosynthesis and oxidative phosphorylation metabolic pathways (e.g., NADH dehydrogenase gene family). This might result from a diverse selective pressure from environment and diverse breeding practice [70]. Similar findings were reported by Cardone et al. [5] and could explain the different adaptation ability to respond to external environmental stresses of one variety with respect to another [5]. A very intriguing case of polymorphic genes within a CNVR was observed on Chr13, where we found members involved in monoterpene (e.g., 3-, 6-hydroxyisobutyryl-CoA hydrolase) and phenylpropanoid (Cinnamyl alcohol dehydrogenase) biosynthetic pathways, as well as operating in the catabolism of fatty acids and certain branched-chain amino acids (e.g., Enoyl-CoA hydratase) [71–73]. In particular, the 3-hydroxyisobutyryl-CoA hydrolase-like protein has been described as a candidate player of terpenes biosynthesis and thus involved in forming floral aromas [74]. Functional annotation of these genes revealed their involvement in the grapevine in Valine-Leucine-Isoleucine degradation and β-alanine metabolism, which produces intermediate compounds involved in aromatic metabolite production. Taken together, these findings suggest the presence of a CNVR on Chr 13 amplified in AGL and FAL (CN doubled or more), which well correlate with differences in the aroma features of these varieties [36, 37, 39–41, 75]. Moreover, data on CNVs shared between AGL and FAL highlighted candidate polymorphic genes related to secondary metabolism, which might explain traits peculiar to these varieties and help their valorization by breeding or technological innovations.

### Interspersed repeats identification and comparative analysis among *V. vinifera* varieties

Transposable elements (TEs) are the most abundant repeated elements in plant genomes [76], impacting genome size and significantly contributing to the plasticity of eukaryotic genomes [77]. We found that 39% of AGL and FAL genomes are composed of TEs, in agreement with Jaillon et al. [58], who reported a similar abundance (41.4%) in the Pinot Noir (PN40024) reference genome. Most TE classes and superfamilies were represented in both genomes, with a large prevalence of LTR-retrotransposons (Class I elements), as observed also in rice, wheat, sunflower, tomato, and potato [50, 78–82]. Using the LTR classification proposed by Neumann et al. [83], seven Gypsy and eight Copia lineages were identified in the genomes of AGL and FAL. He et al. [21], recently obtained similar results by comparing six high-quality grapevine genomes, including *V. vinifera*, *V. sylvestris*, *V. riparia* and *V. amurensis.* This indicates that non-*vinifera* grapes share the same lineages found in the genomes of *vinifera* accessions. However, He et al. [21] pointed out that the Copia superfamily (and particularly *Ale* lineage) was the major component of the LTR retrotransposon in grapevine, in contrast with our results and Velasco et al. [59] that reported a prevalence of Gypsy elements. The discrepancy is probably due to the different approaches used. He et al. [21] identified only intact elements in high-quality genome assemblies, whereas we used an assembly-free approach to identify and quantify TE, which does not distinguish between intact and TE fragments. The prevalence of specific lineages (Athila, Ogre and Ale) could be related to differences in amplification events and insertion site preference of these elements [84]. Interestingly, Jiang and Goertzen [85] found that LTRs were a major cause of the intron expansion in grapes, with a number of Copia-type LTRs about 6.5 times that of Gypsy, in contrast to the predominance of Gypsy in the overall *V. vinifera* genome. However, the genome-wide impact of different LTR lineages on the intraspecific diversity and evolution of *V. vinifera* deserves further analysis.

Since a comprehensive analysis of the *V. vinifera* repeatome composition at lineage level is still lacking in the literature, we performed a comparative similarity-based clustering [53] of low coverage read data in 21 *vinifera* accessions for which the genome is available [9]. The analysis revealed that most clusters of orthologous repeat families contained reads from all accessions, suggesting high conservation of the overall repeatome in terms of TEs types. In addition, the high genomic representation of the Athila and Ale lineages across *Vitis* accessions suggests their predominant role during the *Vitis* divergence. This is particularly intriguing as different studies indicated that genomic amplifications could involve only one or few TE families, significantly contributing to their evolution. For example, 80% of the maize RE repertoire comprises five LTR-REs families [86, 87]. Similarly, approximately 38% of the genome of *Vicia pannonica* is related to a single Ty3-Gypsy-like element [83]. Similar results were also observed in *Arachis* [88] and *Sthylosanthes* [89], where the authors highlighted the preponderance of Athila elements in genomes belonging to the Faboideae subfamily. The variable representation of Ale lineage among grape accessions may have contributed to the diversification of *V. vinifera* genomes, as recently reported by Kwolek et al. [90] in carrot genomes. The mechanisms behind the proliferation of several TE families or lineages are poorly understood, and the most accepted explanation is that these families or lineages lost their cellular silencing mechanisms of the host genome [91, 92]. Our results did not reveal genotype-specific lineages, suggesting a conserved landscape during grapevine evolution. However, clustering analysis disclosed that at least two main clusters of grape cultivars could be identified based on the TE content where multiple TE families appeared either significantly enriched or depleted. Further studies are needed to understand whether these differences can have impacted the host genome and contributed to the diversity within *Vitis* accessions.

### A glimpse into the diversity of the satellite repeats of the grape genome

Our analysis indicated that satDNA accounts for about 3% of FAL and AGL genomes, which is largely consistent with the average estimate obtained from the *vinifera* accessions included here for comparison. However, it also pointed out an almost threefold variation across the grape genomes, with AGL and FAL at the lower end, and Greco Bianco and Pinot at the upper end of the range. Much of this variation was due to different amounts of VvSat1-related repeats, which represented the most abundant satDNA family of FAL and AGL as well as of all the other varieties. This repeat family included several variants that shared high similarity with a candidate centromeric motif identified previously in grapes [33]. Such repeats have been used to predict the centromeres in the grapevine reference genomes [9, 34, 93, 94]. From the analysis of a new Pinot Noir assembly based on long read sequences, Shi at al [34]. found an enrichment of these repeats on each grape chromosome, in regions of few kilobases and up to > 3.5 Mb in length. In addition, the authors detected this repeat family in a single region along most chromosomes but, on a few chromosomes, e.g., Chr16 and 18, it occurred in several locations [34]. Based on this finding, Shi et al. [34] indicated the need for further analysis to elucidate the structure of the centromeric regions in grapes. Here, we provided the first cytological evidence for the association of VvSat1-related repeats with the primary constriction of the grapevine chromosomes. Moreover, the weak signals in some centromeric regions supported the presence of divergent VvSat1 variants and/or other chromosome-specific centromeric sequences [34], as already described in several plant species [26, 95]. However, our FISH analysis did not detect any apparent difference in the pattern and abundance of the VvSat1-related repeats in FAL and AGL compared to Greco Bianco (which had a higher estimate of these repeats). This incongruence between bioinformatic and FISH results may be due to the fact that the clone of Greco Bianco used in the cytological analysis was different from that sequenced. It is also possible that the variation in the abundance of these repeats in AGL/FAL versus Greco Bianco could be below the discrimination level of FISH, especially for detecting strength differences of signals located in highly condensed (peri-) centromeric regions.

Among the satellite repeats with longer monomers, our data suggested that VvSat67 is relatively conserved in the grape genome, since it was located on two chromosome pairs in both FAL and AGL as well as in the other grapes analyzed (in silico and cytologically). Conversely, the number of VvSat214 and VvSat158 sites was polymorphic between FAL and AGL, as well as in comparison to other grapes. Moreover, some VvSat214 and VvSat158 sites were in hemizygosity in AGL and in other varieties. Such variation, including hemizygosity and CNV, is expected in the grape genomes [5, 8, 30]. Indeed, it has been estimated that the two homologous chromosome sets of the Chardonnay reference genome differ between each other by > 15% in length (that is, > 90 Mb), with > 9.0% of this difference due to the repetitive elements that are polymorphic between homologous chromosomes [8]. In addition, a significant portion of the annotated genes is hemizygous in the Chardonnay reference [8]. Hemizygous satellite repeats loci have been reported in both asexually and sexually propagated species [25, 26, 29, 95, 96]. The striking variation of the SatDNA is thought partly related to its rapid turnover [25, 26, 95]. However, it is still unclear how satellite repeats may expand and shrink in the pericentromeric environment, that is the location of VvSat214 hemizygous sites, because the classical mechanisms of unequal crossing-over between tandem arrays are unlikely to occur in recombination-suppressed regions.

## Conclusions

Here we resequenced the genome of two noteworthy grape varieties. A detailed survey of the SNPs, SVs and repetitive elements revealed variations that might contribute to AGL and FAL oenological qualities. In addition, while the overall differences in the repeat composition of AGL and FAL compared to other grapes were relatively small, our data suggested a high diversity among individual insertion sites of TEs and satDNA, including hemyzigousity with presence/absence of specific chromosomal foci and variation in repeat abundance. Further work will determine how these polymorphisms contribute to the distinctive organoleptic and agronomic features of Aglianico and Falanghina.

### Supplementary Information

**Additional file 1:** **Figures S1-S8**

**Additional file 2:** **Supplementary methods**

**Additional file 3:** **Tables S1-S17**

## Data Availability

The raw reads obtained by sequencing the Aglianico and Falanghina genomes were deposited into the Sequence Read Archive (SRA) repository under the BioProject ID: PRJNA1014611, and the reconstructed genome sequences along with their annotations were registered into Mendeley Data Repository with the doi:10.17632/8ftk5snmgy.1.
