# Supplementary material for: Distinct structural variants and repeat landscape shape the genomes of the ancient grapes Aglianico and Falanghina
Source: BMC Plant Biol. 2024 Feb 6;24:88. doi: 10.1186/s12870-024-04778-2 (PMC10845522; doi:10.1186/s12870-024-04778-2)
Supplement: Supplementary file 1 — Additional file 1: Figures S1-S8 [file 12870_2024_4778_MOESM1_ESM.pdf]

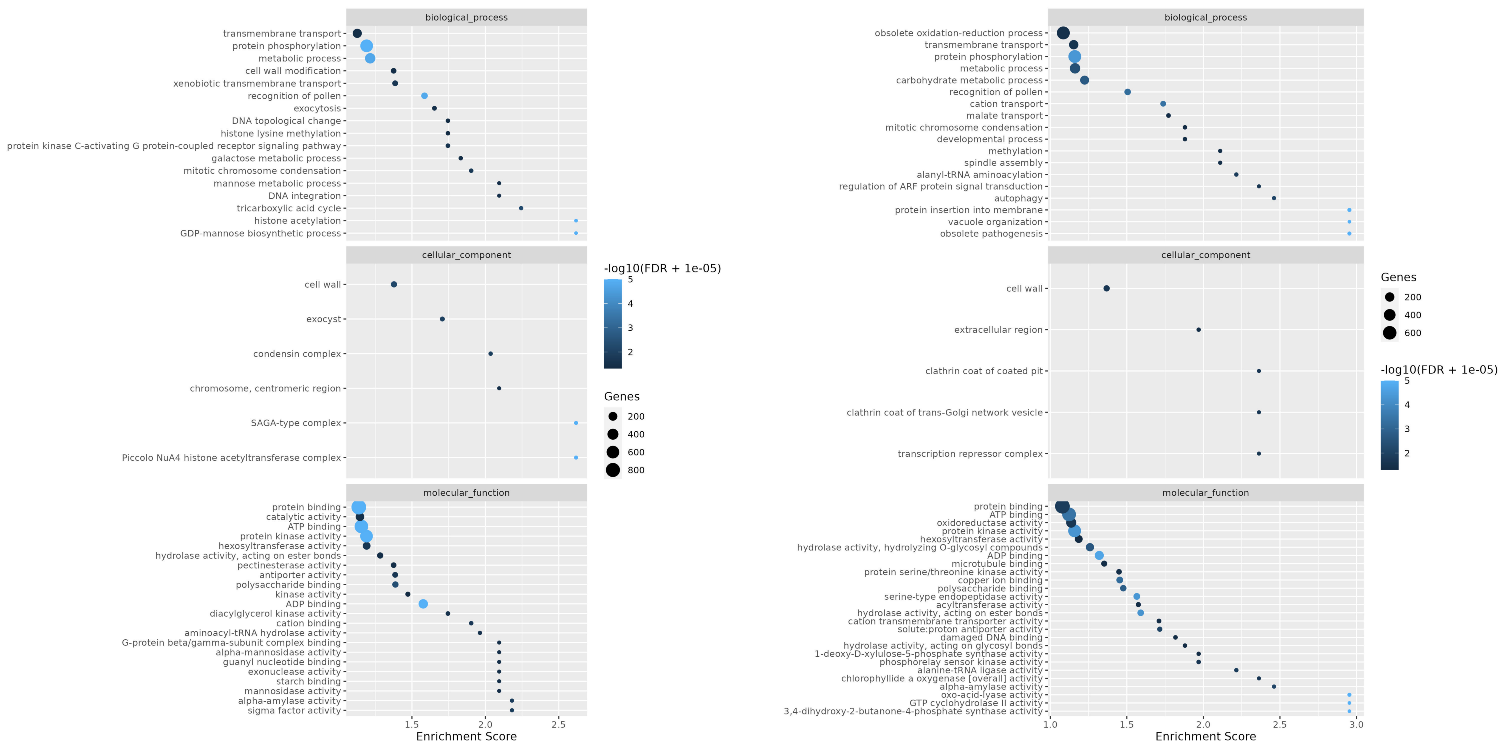

Figure S1

Gene Ontology Enrichment Analysis (GOEA) in Aglianico (left) and Falanghina (right) for genes harboring missense mutations and genes including polymorphisms altering CDS length. Enriched terms related to biological processes, cellular components and molecular functions are reported.

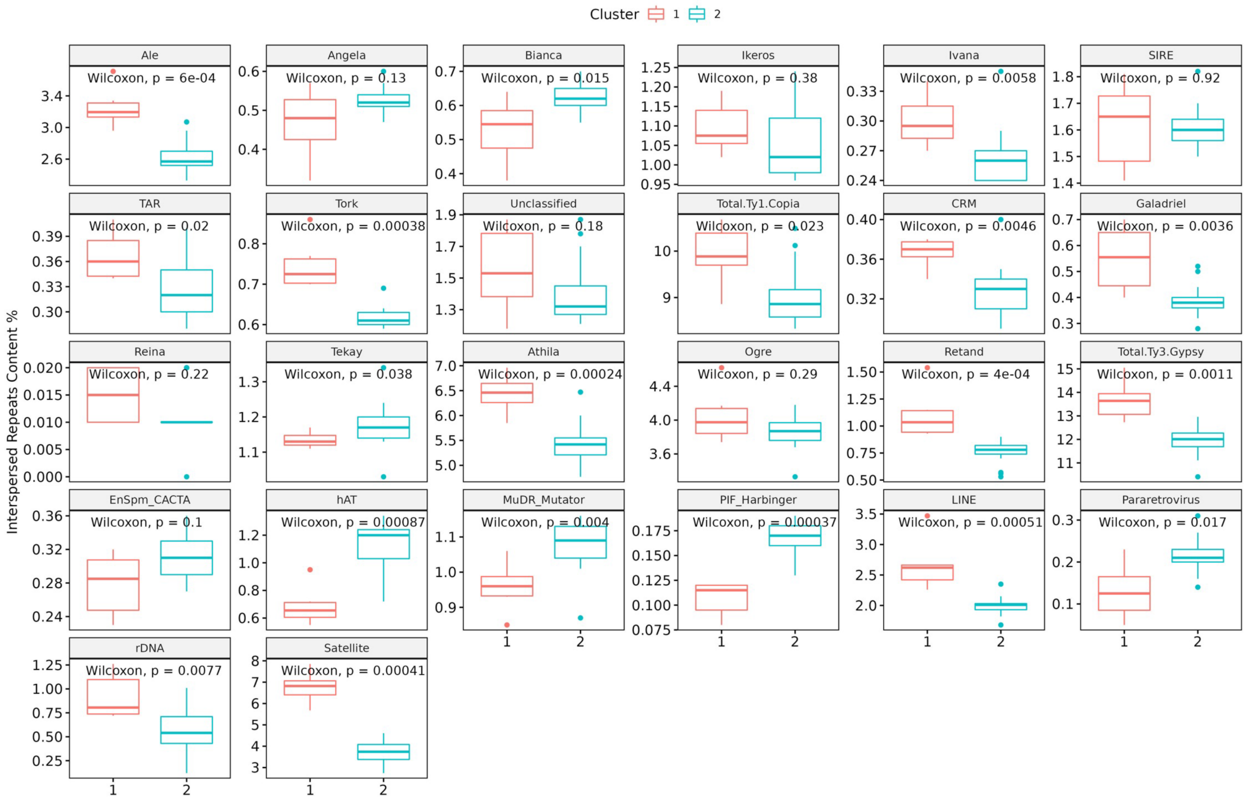

Figure S2

Boxplots showing the interspersed repeats content distribution in two clusters of grape cultivars. Wilcoxon test results are shown for each repeat class

(a)

```
VvSat1      30 CTCACACTGATTCGACCCTTTTTCGGTACTCGTCCGGACAGTTTTTCGAT    79
VvSat21      1 CTCACACTTATTCAACCCTTTTCAGATAGTCGCTAGGACAGTTTTTCGAT    50

VvSat1      80 ACTCGCACGGATTCTACCTTTTCCCGGTACTCGCCCGGATTCTACCATTT   129
VvSat21     51 ACTCGCACGGATTCTGACTTTTTTCGATACTTGCTCGGATTATATCATTT   100

VvSat1     130 TTCGGTACTCACACTGATTCGACCCTTTTTCGGTACTCGTCCGGACAGTT   179
VvSat21    101 TTCGGTACTCACACTTATTCGACTCTTTTCTGGTACTCGCTCGCACAGTT   150

VvSat1     180 TTTCGATACTCGCACGGATTCTACCTTTTCCCGGTACTCGCCCGGATTCT   229
VvSat21    151 TTTCGATACTCACACGGATTCT----------------------------   172

VvSat1     230 ACCATTTTTCGGTACTCACACTGATTCGACCCTTTTTCGGTACTCGTCCG   279
VvSat21    173 AACATTTTTCAGTAATCACACTTATAAGACGTTTATACAGAAGACGCCCG   222

VvSat1     280 GACAGTTTTTCGATACTCGCACGGATTCTACCTTTTCCCGGTACTCGCCC   329
VvSat21    223 GACTATTTTTCGATAATAACACGTATTCTGCCATTTCTCGGTACTCCCCC   272

VvSat1     330 GGATTCTACCATTTTTCGGTA    350
VvSat21    273 GAATTCTATCATTTTTCAATA    293
```

(b)

```
VvSat1      25 CGGTACTCACACTGATTCGACCCTTTTTCGGTACTCGTCCGGACAGTTTT    74
VvSat141R    2 CGGTACTCACAAAGATTCTACC-TTTTCCGGTACTCGCCCGGACAGTTTT    50

VvSat1      75 TCGATACTCGCACGGATTCTACCTTTTCCCGGTACTCGCCCGGATTCTAC   124
VvSat141R   51 TCGGTACTCTCACGGATTCTACATATTCCCGGCACTCGCACGGATTCTAC   100

VvSat1     125 CATTTT    130
VvSat141R  101 CGTTTT    106
```

(c)

```
VvSat1      60 CGTCCGGACAGTTTTTCGATACTCGCACGGATTCTACCTTTTCCCGGTAC   109
VvSat159     1 CGTCCGGACACTTTTTCGGTACTCGGACGGATTCTAGCTTTTCCCGATAC    50

VvSat1     110 TCGCCCGGATTCTACCATTTTTCGGTACTCACACTGATTCGACCCTTTTT   159
VvSat159    51 TGAACCGGATTGTACCATTTTGCGGTACTCACACTGATTGTACCCTCTTC   100

VvSat1     160 CGGTACT    166
VvSat159   101 CGATACT    107
```

Figure S3

Pairwise sequence alignment between consensus monomers of VvSat1 and (a) VvSat21, (b) the reverse complement of VvSat141 (VvSat141R), (c) VvSat159. Identities are shaded in light blue.

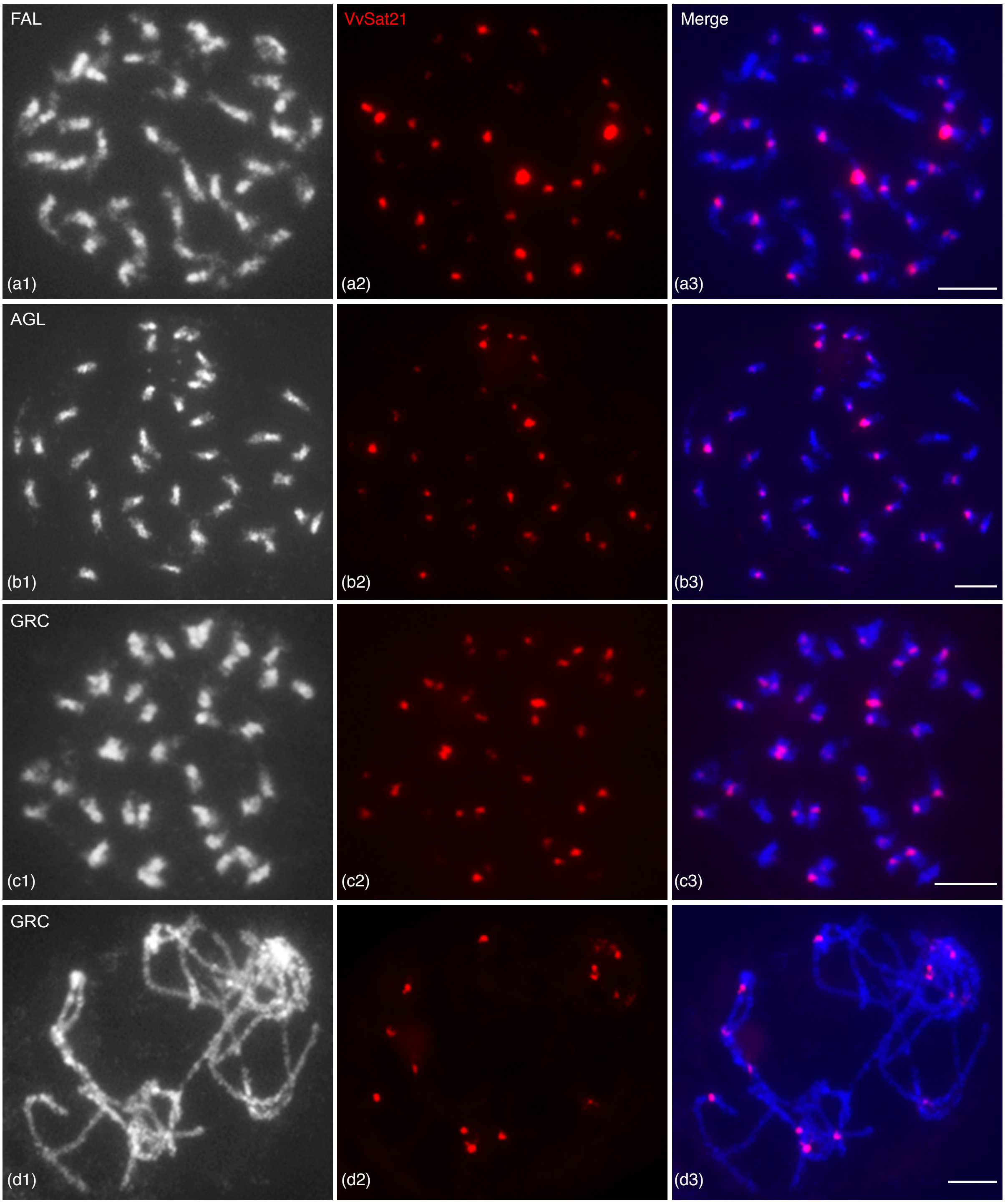

Figure S4

FISH localization of VvSat1-related repeats (VvSat21) on the grape chromosomes. First column: Somatic metaphase chromosomes of Falanghina (FAL, a1), Aglianico (AGL, b1), and Greco Bianco (GRC, c1), and meiotic pachytene chromosomes of Greco Bianco (GRC, d1). Middle column: FISH signals derived from VvSat21. Third column: Merged images of the chromosome spreads (first column) and their related FISH signals (middle column). Scale bars = 5 μm.

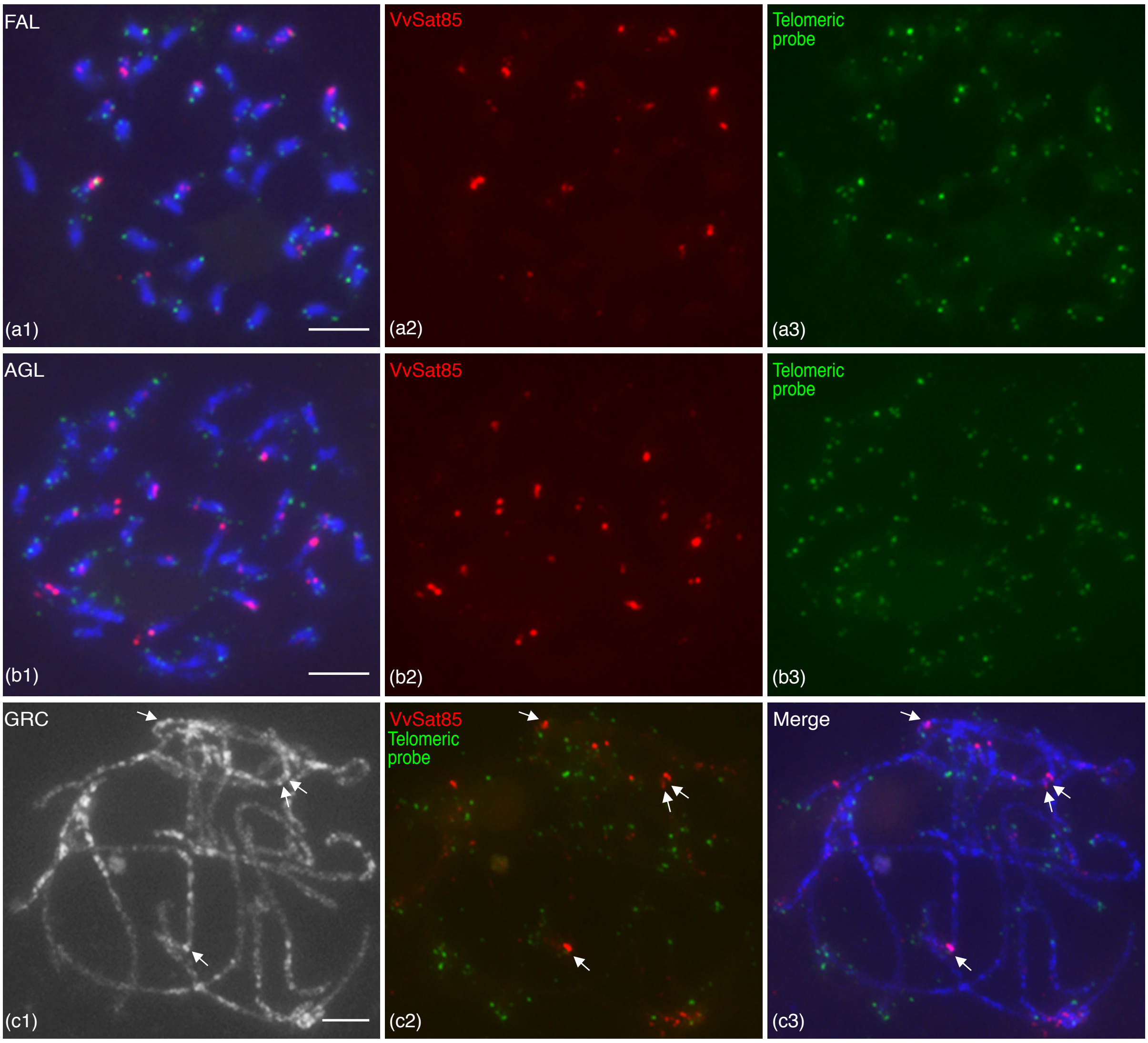

Figure S5

FISH mapping of VvSat85 (red) and a telomeric repeat (TTTAGGG)4 probe (green) on the mitotic chromosomes of Falanghina (FAL, a1-a3) and Aglianico (AGL, b1-b3), and on the pachytene chromosomes of Greco Bianco (GRC, c1-c3). (c1) Greco Bianco pachytene chromosomes are shown as a black-and-white image to enhance the visualization of heterochromatin. Arrows in c1 point to the heterochromatic domains that are overlapped by VvSat85 signals. Scale bars = 5 µm.

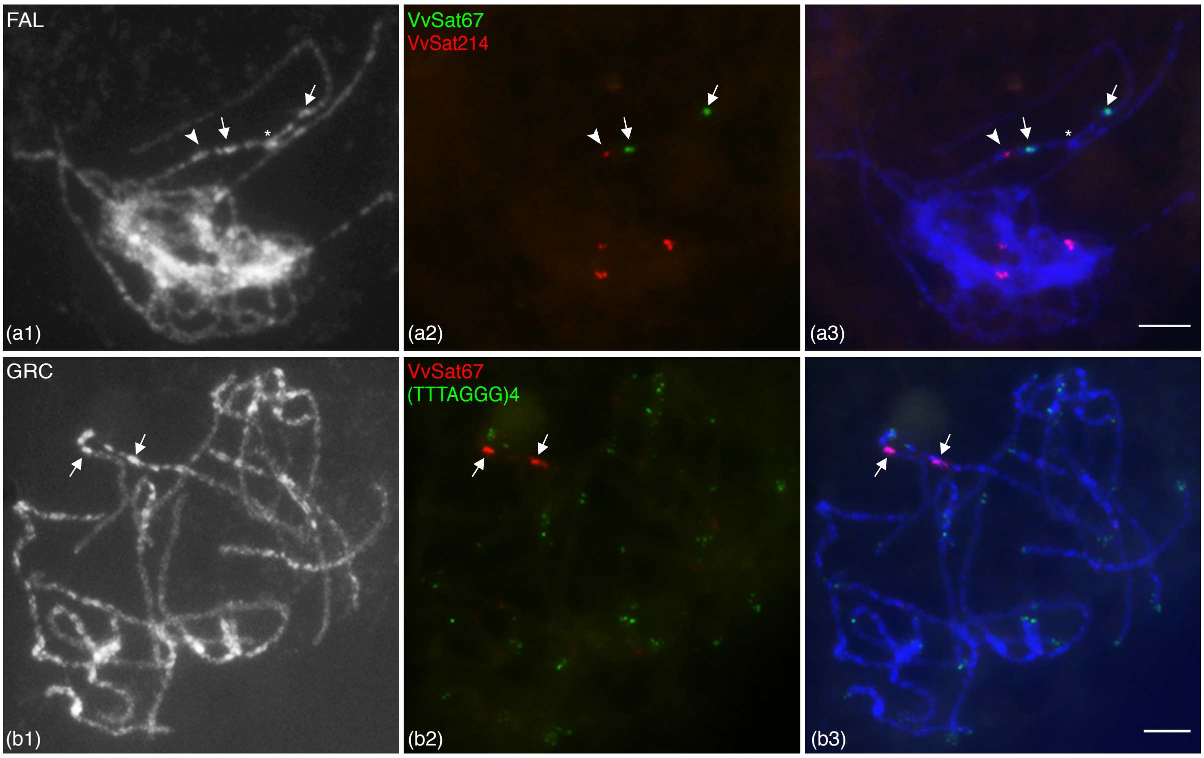

Figure S6

FISH localization of VvSat67 repeats in the pericentric heterochromatin of two chromosome pairs of grapevine. (a1) Pachytene chromosomes of Falanghina (FAL) shown as a black and white image to enhance the visualization of the heterochromatin; (a2) FISH signals derived from VvSat67 (green) and VvSat214 (red) repeats; (a3) merged image from (a1) and (a2). (b1) Pachytene chromosomes of Greco Bianco (GRC) grape; (b2) FISH signals derived from VvSat67 (red) and a telomeric repeat (TTTAGGG)4 probe (green); (b3) Merged image of (b1) and (b2). Arrows point to the heterochromatic domains overlapped by VvSat67 signals; the arrowhead in (a1-3) indicates the VvSat214 site co-located on the same chromosome carrying a VvSat67 site (possibly grape chr. 15); the asterisk in (a1, a3) indicates the overlapping ends between the chromosomes carrying VvSat67 sites. Scale bars = 5 μm.

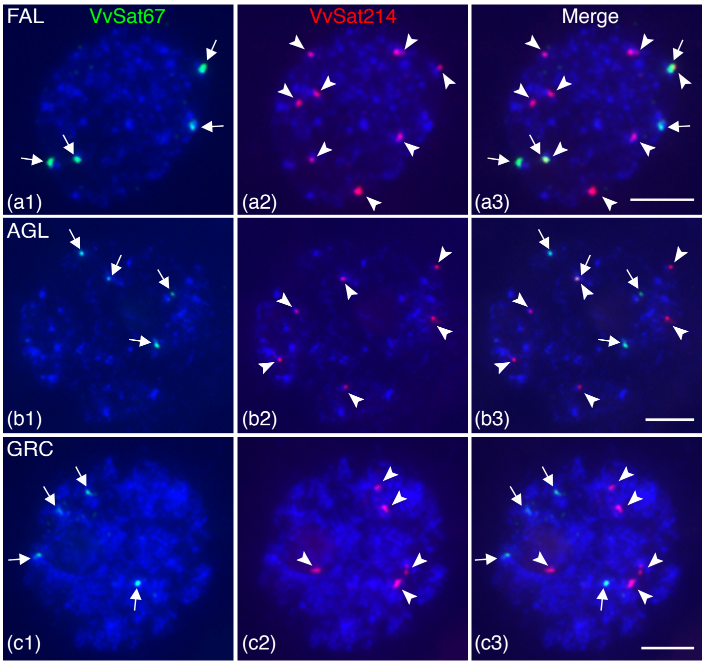

Figure S7

FISH using VvSat67 (green signals, arrows) and VvSat214 repeats (red, arrowheads) on the nuclei of the grapes Falanghina (FAL, a1-3), Aglianico (AGL, b1-3) and Greco Bianco (GRC, c1-3). Scale bars = 5 μm.

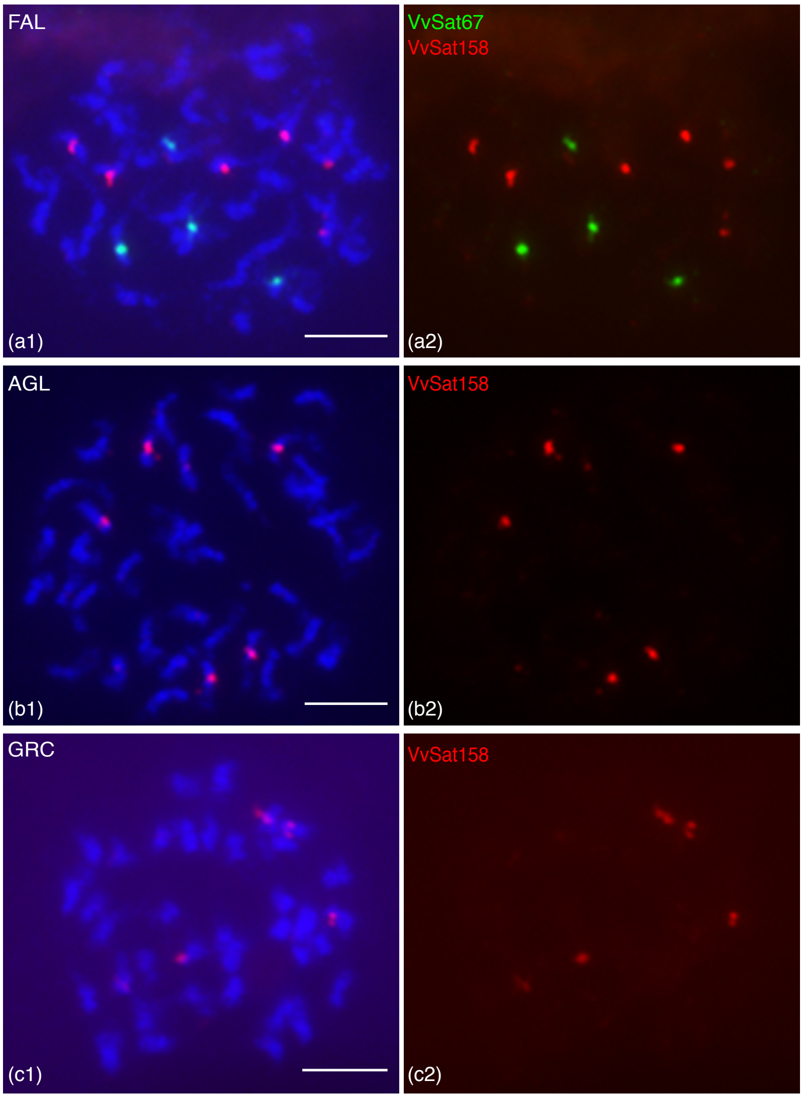

Figure S8

Localization of VvSat158 repeats (red signals) on the mitotic chromosomes of the grapes Falanghina (FAL, a1), Aglianico (AGL, b1) and Greco Bianco (GRC, c1) by FISH. (a1-a2) FISH signals generated by VvSat158 (red) and VvSat67 (green) repeats in FAL. None of the VvSat158 signals co-localized with VvSat67. (b2-c2) FISH signals generated by VvSat158 (red) in AGL (b2) and GRC (c2). Scale bars = 5 μm.
