# Supplementary material for: Distinct structural variants and repeat landscape shape the genomes of the ancient grapes Aglianico and Falanghina
Source: BMC Plant Biol. 2024 Feb 6;24:88. doi: 10.1186/s12870-024-04778-2 (PMC10845522; doi:10.1186/s12870-024-04778-2)
Supplement: Supplementary file 2 — Additional file 2: Supplementary methods [file 12870_2024_4778_MOESM2_ESM.docx]

**SUPPLEMENTARY METHODS**

*Reference guided assembly*

Reference-guided assembly was performed using Reconstructor pipeline (v1.0) (<http://www.sequentiabiotech.com/omicstools/pipeline/>, October 2017), combined with the *Pinot noir* 12X reference genome (https://urgi.versailles.inra.fr/Species/Vitis/Data-Sequences/Genome-sequences). A two-step strategy was used: iterative read mapping and *de novo* assembly of unmapped reads. In the first step, high-quality reads were aligned against the reference genome using SUPER-W (10.1093/molbev/msv152). Five iterations were performed to identify sequence variations such as SNPs, deletion and insertion polymorphisms (DIPs), and structural variations (SVs). At each iteration, only the variants with a genotype quality (GQ) higher than 30, coverage higher than 6x and an allele frequency higher than 0.75 were kept. The identified polymorphisms were used to edit the grape reference genome using VCF tools (v. 0.1.12 b, 10.1093/ bioinformatics/btr330), thus obtaining cultivar-specific sequences. The second step based on the *de novo* assembly of the reads that did not map on the reference genome was performed by using SOAPdenovo (v2.04, 10.1186/2047-217X-1-18) setting two k-mer sizes: 50 and 120 nt. Resulting contigs were filtered for size (> 400 bp) and subjected to BLASTN (v2.2.30þ) search (e-value 0.01) against the NCBI non-redundant nucleotide database section ‘Viridiplantae’ in order to remove possible contaminants (Table S16 and S17). Using paired-read and split read approaches, Reconstructor was employed to combine the genotype-specific reconstructed genomes with *de novo* assembled contigs in order to obtain final private genome sequences. At least four paired-end reads covering the junction between the reference chromosomes and the *de novo* assembled contigs were required to generate new insertions. Low-complexity sequences, repetitive sequences and interspersed repeats within the reconstructed genomes were identified and classified using RepeatMasker (v4.0.5). The regions highlighted from the assemblies of Aglianico and Falanghina as deletions or insertions were localized in the reference genome (12X) and classified as belonging to repetitive, intergenic or genic regions. The dataset used for the reconstruction of Aglianico (AGL) and Falanghina (FAL) genome sequences included 33.7 M and 40.9 M of 30-150 bp paired-end reads, respectively. Gene Ontology Enrichment Analysis (GOEA) was performed on the genes showing missense mutations and genes with polymorphisms altering CDS length (i.e., disruptive in-frame deletions, disruptive in-frame insertions, frameshift variants, stop coding gain/loss and start codon loss). GOEA was performed with in-house scripts and was based on a hypergeometric test comparing the proportion of genes in each GO category in the genome and the groups of mutated genes; an FDR ≤ 0.05 was considered significant.

*RNAseq analysis*

The AGL and FAL transcriptome data were previously published by some authors of this manuscript and recently published by Villano et al. [1]. Briefly, samples were collected from plants arranged in a completely randomized experimental design with three biological replicates and ten vines per replicate. For each biological replicate, approximately 200 berries were harvested at pre-*veraison* (PV), *veraison* (V) and ripening (R) stages as reported in Fasoli et al. [2], immediately frozen in liquid nitrogen at the time of collection and then stored at –80°C. In the laboratory, skins and pulps were separated from frozen berries, homogenized to produce a fine powder and used for RNA extractions and metabolite determinations. For each sample, total RNA was isolated from berries tissues as described by Japelaghi et al. [3] with few modifications. RNA concentrations were determined using a NanoDrop ND-1000 spectrophotometer (Thermo Scientific, Wilmington, USA) and its integrity was verified using a bioanalyzer (Agilent Technologies, Santa Clara, California, USA). Thirty-six cDNA libraries (three biological replicates from pulp and skin from three developmental stages for both varieties) were produced by Genomics4Life srl (Salerno, Italy, http://www.genomix4life.com) from three micrograms of total RNA and subsequently sequenced using the Illumina HiSeq 2500 sequencing platform, providing a total of approximately 70M read/sample. Before further analysis, a quality check was performed on the raw sequencing data using FastQC. Low-quality reads were removed with BBDuk and after trimming, the minimum length of the reads was set to 35 bp and the minimum base quality score to 25. High-quality reads were aligned against the *V. vinifera* cv. Falanghina and cv. Aglianico references genome sequences using the STAR aligner (version 2.5.0c). To rescue the multiple mapping reads, expression levels were quantified with RSEM (version 1.2.31) after mapping the reads against the transcriptome of both varieties. Gene expression amounts were normalized by calculating the Target Fragment Per Kilobases Per Million Reads (FPKM) value. All the statistical analyses were performed with R with the package EBSeq and EBSeq-HMM.

*Genome annotation*

Details regarding AGL and FAL genome annotation statistics are reported in Table S1. Regarding genome annotation, we first assembled the Aglianico and Falanghina transcripts (including splicing isoforms) from custom RNA-seq data. Reads from Aglianico and Falanghina fruits were subjected to normalization using Trinity (v2.0.6, 10.1038/nbt.1883). Then, normalized reads were aligned on the corresponding reconstructed genome sequences with STAR (v. 2.4.2a, 10.1093/bioinformatics/bts635). The resulting BAM files were filtered to remove duplicates with Picard MarkDuplicates (v. 1.31, http://broadinsti tute.github.io/picard/ (October 2017)) and then used as input for Trinity (v2.0.6) to perform genome-guided transcriptome assembly. In order to remove redundancy, transcripts were postprocessed using the CD-HIT-EST (v4.6, 10.1093/bioinformatics/ btl158) tool, which clusters similar transcripts based on a similarity threshold (99% of identity). Trinity assembled transcripts and the official grape gene models were merged and fed into the PASA genome annotation pipeline (v.2.0.2, 10.1093/nar/gkg770). The annotation was fully post-processed through different filtering steps to reduce false positives most likely due to assembly errors. Firstly, a transcript-level quantification was carried out using eXpress (v1.5.1, 10.1093/bioinformatics/btt034). Those splicing variants with an expression level < 15% compared to the most expressed isoform were removed due to their high probability of being assembly artifacts. In addition, single exon sequences overlapping multi-exon transcripts on the same strand were filtered out as they were also considered mis-assemblies. To classify the predicted transcripts, a lncRNA prediction analysis was carried out following the approach described by Paytuvı-Gallart and collaborators (https://academic.oup.com/nar/article/44/D1/D1161/2502695). Only the coding transcripts were further analysed using TransDecoder (v2.0.1, 10.1038/nprot.2013.084) in order to identify candidate coding regions (CDS) and untranslated regions (UTRs). CDS were functionally annotated using InterProScan (v. 5, 10.1093/bioinformatics/ btu031). In addition, BLASTP (v2.2.30þ) searches were performed against the *Arabidopsis thaliana* (TAIR10) protein complement. Only those hits with an e-value of 0.001 and a percentage match length > 20% were considered.

*Repetitive sequences analysis*

The repeated fraction was evaluated by graph-based clustering of repetitive elements in unassembled reads using the RepeatExplorer2 Web server [4, 5]. Due to the technical limitations of RepeatExplorer2 [4, 5] (*e.g.,* server memory = 64 Gb RAM), we used available raw paired-end Illumina datasets of 21 grapevine genotypes from Magris et al. [6], which were selected to capture the highest genotypic diversity in the dataset (SRR6156270, SRR6156387, SRR6156386, SRR6156407, SRR5627798, SRR6156422, SRR6156280, SRR6156332, SRR6156412, SRR6156309, SRR5627781, SRR5627793, SRR3990781, SRR6156316, SRR6156291, SRR6156331, SRR6156294, SRR5627799, SRR13343627, SRR6156274, SRR6156317, SRR6156276, SRR6156405, SRR924196) plus the Aglianico and Falanghina raw reads of the present study. The sequences were obtained through the “European Nucleotide Archive” (EBI) database. Seqtk (<https://github.com/lh3/seqtk>) was used to extract 1 M random reads (seed 100) from each sample. Adapter removing and read quality analysis were performed with Trimmomatic (v0.39) [7] to trim bases with a quality score (QS) < 20, remove reads < 100nt, and cut reads to 100nt to obtain a subset of high-quality reads of the same length for each sample (100nt). Two different strategies were used for the analysis of repetitive sequences. The first consisted of independent clustering analyses using a set of 250,000 high-quality random reads of Aglianico and Falanghina, covering the 0.01 × of their genomes, whereas the second comprised a comparative analysis using the concatenated dataset (250,000 random reads for each sample). In both scenarios, the clustering was performed using the default settings of 90% similarity over 55% of the read length. After removing clusters classified as plastid and contamination, the remaining top clusters were manually screened to refine ambiguous repeats using two approaches. The first consisted in inspecting the annotation of each cluster belonging to ambiguous annotated superclusters (i.e., ‘repeat’). If the ambiguous annotation was due to conflicting evidence pointing to different types of repetitive elements, the supercluster was split into groups of clusters that could be consistently annotated as single repetitive elements. The second approach was to inspect the contigs assembly file using tablet (https://ics.hutton.ac.uk/tablet/) to search features which may indicate a specific type of repeat (i.e., mobile element insertion sites). Finally, to estimate the genomic proportion, we first calculated the number of reads representing only the nuclear sequences NN as: NN = NA - NO - NC, where NA is the number of analyzed reads, NO is the number of reads annotated as plastid or mitochondrial DNA and NC is the number of reads from contamination. Then, we calculated the genomic proportion of individual clusters by dividing the size (number of reads) of the clusters by NN, and finally, we summed the genomic proportions of each cluster assigned to the same repeat type. Principal component analysis based on the transposon content was performed with the R in-built function prcomp, and the plot was generated with ggplot2. Clustering was performed with the k-means function after identifying the best number of clusters using the package clValid using Euclidean distance as metric and stability and internal indexes for the evaluation. The optimal number of clusters was automatically defined based on the optimal scores of Connectivity, Silhouette, APN and ADM. The TE family distribution in the observed clusters was plotted using ggpubr and performing the Wilcoxon test.

*Analysis and validation of selected satellites*

The putative satellite repeats were checked for possible similarities with sequences deposited in Repbase using Censor (<http://www.girinst.org/censor>). Similarities between the satellite repeats were detected by all-against-all BLAST searches. Pairwise alignments between VvSat1 monomers and other satellites were performed using the program matcher available online from Emboss package (https://www.bioinformatics.nl/cgi-bin/emboss). In the pairwise alignments, we used multimers of VvSat1 (instead of a single monomer), because tandem repeat boundaries are arbitrary, which makes it possible for related repeats to align in a staggered fashion and over only a fraction of their true length (Figure S3). In order to gain support for their tandem arrangement, the “atypical” long monomers of VvSat67, VvSat214 and VvSat158 were BLAST searched against the V. vinifera reference genomes of PN40024_12X.v2 and Cabernet Sauvignon clone 08_v1.1 (available at <http://www.grapegenomics.com/pages/PN40024/> and <http://www.grapegenomics.com/pages/VvCabSauv/>) using default parameters. Blast results with the following parameters were retained: at least 80% identity with the motif query, alignment over at least 80% of the motif length, at least 10 adjacent/close hits (Tables S14,and 15). VvSat67 mapped on the pseudomolecules of chr-15 and 17 of both reference genomes (Tables S14,and 15). VvSat214 monomers were located on the pseudomolecules of chr-10, 11, 15 and 16 of PN40024 (Table S14). However, on CabSauv08 reference, VvSat214 mapped on three pseudomolecules of the haplotype 1 (Chr-10, 11 and 19) and four pseudomolecules of haplotype 2 (Chr-10, 11, 15, and 16). Finally, VvSat158 mapped to chr-10, 13, 15 and 16 of PN40024; whereas, on CabSauv08 reference, it mapped to chr-10 and 15 of both haplotypes, plus on chr-16 of the haplotype 2 (Table S15). The distribution of selected putative satellites on the grape chromosomes was assessed by fluorescence in situ hybridization (FISH). Oligonucleotide probes and PCR primers for FISH were designed on their consensus monomers (Data S1). The oligoprobes were labeled on the 5’ end with either fluorescein or Tamra during synthesis (GenScript Biotech, Leiden, The Netherlands). PCR for repeat amplification (from Aglianico or Falanghina genomic DNA) was performed in 50 μl of reaction [(10-20 ng of template DNA, 1× PCR buffer, 3 mM MgCl2, 0.2 mM dNTPs, with 0.2 μM primers, 0.5 U of Taq polymerase (Invitrogen)] for 34 cycles of 30 sec at 95°C, 20 sec at 60 °C, 60 sec at 72°C, preceded by initial denaturation (4 min at 95°C) and followed by a final extension step (5 min at 72°C). Probes were labeled by standard nick-translation reaction with either digoxigenin-11-dUTP or biotin-16-dUTP (Roche Diagnostics, Indianapolis, Indiana). Immature flower buds of Aglianico, Falanghina and Greco Bianco were fixed in 3:1 (100% ethanol: glacial acetic acid) Carnoy’s solution. Mitotic and meiotic chromosome preparation and FISH were performed as previously described [8, 9]. A minor modification was that the dissected anthers were incubated in the enzyme mix (5% cellulase Onozuka R10, 2% pectinase, 0.3% pectolyase Y23, 0.2% cytohelicase) for 6-8 hours. Digoxigenin and biotin-labeled probes were detected using anti-digoxigenin conjugated rhodamine antibody (Roche Diagnostics) and Alexa Fluor 488 streptavidin (Thermo Fisher), respectively. Chromosomes were counterstained with 4′,6-diamidino-2-phenylindole (DAPI) in Vectashield antifade medium (Vector Laboratories, Burlingame, CA, USA). Images were captured with a DFC365 FX CCD camera and LAS AF software attached to a Leica DM6000B epifluorescence microscope (Leica Microsystems). The final contrast of the images was adjusted in Adobe Photoshop.

*Genome-wide identification and variants analysis of genes involved in the biosynthesis of secondary metabolites*

The key genes of the pathways of terpenes, green leaf volatiles (GLVs), BCAA (branched-chain amino acids), and phenylpropanoids were identified as reported in Esposito et al. [10] and Villano et al. [11]. The proteins used as queries to search for amino acid orthologs in Aglianico and Falanghina genomes were obtained from *A. thaliana* for *geranyl geranyl pyrophosphate synthase (GGPPS), alcohol acetyl transferase (AACT), 3-hydroxy-3-methylglutaryl-coa synthase (HMGS), 3-hydroxy-3-methylglutaryl-coa reductase (HMGR), mevalonate kinase (MVK), 5-phosphomevalonate kinase (PMK), mevalonate 5-diphosphate decarboxylase (MVD)*, and from *V. vinifera* PN40024 12X.v2 assembly for *1-Deoxy-D-xylulose 5-phosphate synthase (DXS), 1-deoxy-d-xylulose 5-phosphate reductoisomerase (DXR), 4-diphosphocytidyl-2-C-methyl-D-erythhtol synthase (MCT), 4-(cytidine 5′-diphospho)-2-C-methyl-D-erythritol kinase (CMK), (E)-4-hydroxy-3-methylbut-2-enyl diphosphate reductase (HDR)* and *terpene synthase* (*TPS*). The protein sequences related to the remaining genes involved in phenylpropanoids and terpenes were collected from Savoi et al. [12].

**References**

1. Villano, C., Demurtas, O. C., Esposito, S., Granell, A., Rambla, J. L., Piombino, P., et al. (2023). Integrative analysis of metabolome and transcriptome profiles to highlight aroma determinants in Aglianico and Falanghina grape berries. BMC Plant Biology, 23(1), 1-15.

2. Fasoli, M., Dal Santo, S., Zenoni, S., Tornielli, G. B., Farina, L., Zamboni, A., et al. (2012). The grapevine expression atlas reveals a deep transcriptome shift driving the entire plant into a maturation program. The Plant Cell, 24(9), 3489-3505.

3. Japelaghi, R. H., Haddad, R., & Garoosi, G. A. (2011). Rapid and efficient isolation of high-quality nucleic acids from plant tissues rich in polyphenols and polysaccharides. Molecular biotechnology, 49(2), 129-137.

4. Novák, P., Neumann, P., Pech, J., Steinhaisl, J., & Macas, J. (2013). RepeatExplorer: a Galaxy-based web server for genome-wide characterization of eukaryotic repetitive elements from next-generation sequence reads. Bioinformatics, 29(6), 792-793.

5. Novák P, Neumann P, Macas J. Global analysis of repetitive DNA from unassembled sequence reads using RepeatExplorer2. Nature Protocols. 2020;15(11):3745-3776.

6. Magris, G., Jurman, I., Fornasiero, A., Paparelli, E., Schwope, R., Marroni, F., et al. (2021). The genomes of 204 Vitis vinifera accessions reveal the origin of European wine grapes. Nature communications, 12(1), 1-12.

7. Bolger, A. M., Lohse, M., & Usadel, B. (2014). Trimmomatic: a flexible trimmer for Illumina sequence data. Bioinformatics, 30(15), 2114-2120.

8. Braz, G. T., He, L., Zhao, H., Zhang, T., Semrau, K., Rouillard, J. M., et al. (2018). Comparative Oligo-FISH Mapping: An Efficient and Powerful Methodology To Reveal Karyotypic and Chromosomal Evolution. *Genetics*, *208*(2),513–523.

9. Iovene, M., Cavagnaro, P. F., Senalik, D., Buell, C. R., Jiang, J., & Simon, P. W. (2011). Comparative FISH mapping of Daucus species (Apiaceae family). *Chromosome research 19*(4), 493–506.

10. Esposito, S., Aversano, R., D’amelia, V., Villano, C., Alioto, D., Mirouze, M., et al. (2018). Dicer-like and RNA-dependent RNA polymerase gene family identification and annotation in the cultivated Solanum tuberosum and its wild relative S. commersonii. Planta, 248(3), 729-743.

11. Villano, C., Esposito, S., D’Amelia, V., Garramone, R., Alioto, D., Zoina, A., et al. (2020). WRKY genes family study reveals tissue-specific and stress-responsive TFs in wild potato species. Scientific reports, 10(1), 1-12.

12. Savoi, S., Wong, D. C., Arapitsas, P., Miculan, M., Bucchetti, B., Peterlunger, E., et al. (2016). Transcriptome and metabolite profiling reveals that prolonged drought modulates the phenylpropanoid and terpenoid pathway in white grapes (Vitis vinifera L.). BMC plant biology, 16(1), 1-17.
